# Multi-Omics Analyses Detail Metabolic Reprogramming in Lipids, Carnitines, and Use of Glycolytic Intermediates between Prostate Small Cell Neuroendocrine Carcinoma and Prostate Adenocarcinoma

**DOI:** 10.3390/metabo9050082

**Published:** 2019-04-26

**Authors:** Bei Gao, Hui-Wen Lue, Jennifer Podolak, Sili Fan, Ying Zhang, Archana Serawat, Joshi J. Alumkal, Oliver Fiehn, George V. Thomas

**Affiliations:** 1 West Coast Metabolomics Center, University of California, Davis, CA 95616, USA; begao@ucsd.edu (B.G.); slfan@ucdavis.edu (S.F.); ythzhang@ucdavis.edu (Y.Z.); 2 Knight Cancer Institute, Oregon Health and Science University, Portland, OR 97239, USA; lue@ohsu.edu (H.-W.L.); podolakj@ohsu.edu (J.P.); sehrawat@ohsu.edu (A.S.); alumkalj@ohsu.edu (J.J.A.); 3 Department of Medicine, Oregon Health and Science University, Portland, OR 97239, USA; 4 Department of Pathology and Laboratory Medicine, Oregon Health and Science University, Portland, OR 97239, USA

**Keywords:** oxygen consumption rate, extracellular acidification rate, hydrophilic interaction liquid chromatography (HILIC), *N-Myc*

## Abstract

As the most common cancer in men, prostate cancer is molecularly heterogeneous. Contributing to this heterogeneity are the poorly understood metabolic adaptations of the two main types of prostate cancer, i.e., adenocarcinoma and small cell neuroendocrine carcinoma (SCNC), the latter being more aggressive and lethal. Using transcriptomics, untargeted metabolomics and lipidomics profiling on LASCPC-01 (prostate SCNC) and LNCAP (prostate adenocarcinoma) cell lines, we found significant differences in the cellular phenotypes of the two cell lines. Gene set enrichment analysis on the transcriptomics data showed 62 gene sets were upregulated in LASCPC-01, while 112 gene sets were upregulated in LNCAP. ChemRICH analysis on metabolomics and lipidomics data revealed a total of 25 metabolite clusters were significantly different. LASCPC-01 exhibited a higher glycolytic activity and lower levels of triglycerides, while the LNCAP cell line showed increases in one-carbon metabolism as an exit route of glycolytic intermediates and a decrease in carnitine, a mitochondrial lipid transporter. Our findings pinpoint differences in prostate neuroendocrine carcinoma versus prostate adenocarcinoma that could lead to new therapeutic targets in each type.

## 1. Introduction

Prostate cancer is diagnosed in over 160,000 men annually in the United States, making it the most common cancer in men. Androgen receptor signaling is the primary driver of prostate cancer, and subsequently, medical castration with androgen deprivation therapy is the backbone of all treatments in men with metastatic prostate cancer [1,2]. Estrogens may also play a role in the pathogenesis of prostate cancer [3]. Estrogen effects are mediated by estrogen receptor α (ERα) and β (ERβ). ERα acts as an oncogene, while ERβ plays an anti-oncogenic role [3]. Selective ER modulators such as tamoxifen and raloxifene induce apoptosis in the androgen-sensitive human prostate cancer cell line LNCAP, which express ERβ but not ERα [4,5]. Taken together, this suggests crosstalk between androgen and estrogen receptors in prostate cancer. Clinically, most men are diagnosed with adenocarcinoma of the prostate, with a minor subset (<1%) developing small cell neuroendocrine prostate cancer (SCNC) [6]. However, we noted SCNC is increasingly seen in men with castration-resistant prostate cancer who have been treated with newer androgen signaling inhibitors [6]. Patients with SCNC have limited treatment options. A small component of neuroendocrine cells is present among prostate-specific antigen (PSA)-producing luminal cells and basal cells, but it represents less than 1% of cells in the epithelia compartment of the prostate. However, it is unclear at present what the cell of origin is in prostate adenocarcinomas that become neuroendocrine carcinomas under the selective pressure of androgen deprivation treatments. Understanding the specific metabolic needs of different prostate cancer types holds promise for the discovery of potential druggable targets and development of prostate cancer treatment strategies.

Metabolic reprogramming in cancer cells has been recognized as a hallmark of cancer [7]. Many cancer cells exhibit a shift to increased aerobic glycolysis and lactate production, known as the Warburg effect [8]. However, prostate tumors mostly show divergent metabolic phenotypes that may further change during tumor pathogenesis and, consequently, the Warburg effect may only partially meet their metabolic demands [9]. Glucose metabolism in a SCNC cell line PC-3 in comparison with a prostate adenocarcinoma cell line LNCAP was investigated, and glycolytic features of PC-3 cells were higher than those of LNCAP cells [10]. However, other metabolic pathways in SCNC have not been examined previously, and research on comprehensive metabolic profiling of SCNC is lacking. More detailed knowledge of metabolic changes between stages and types of prostate cancers will help to identify new drug targets and therapeutic strategies. 

Normal prostate epithelial cells have a distinct metabolic profile by exporting citrate from mitochondria to the cytoplasm and then excreting citrate as a major component of prostatic fluid [11]. In this way, mitochondrial oxidative phosphorylation at baseline is limited while still utilizing pyruvate dehydrogenase instead of exporting lactate. Prostate cells have a very high rate of production and excretion of citrate in comparison to other organs [12,13], giving the prostate peripheral zone epithelium a very distinct metabolic phenotype [14]. Yet, when prostate cells experience neoplastic transformation, profound metabolic alterations occur, including the metabolic transformation from citrate accumulation to citrate oxidation [15]. Increased citrate oxidation in prostate cancer cells results in more efficient energy production and supports high cellular energy demand [13]. In order to maintain elevated citrate oxidation, alterations in fatty acid metabolism have been suggested [13].

To study these metabolic differences between two prostate cancer types in detail, we performed untargeted metabolomics, lipidomics, and transcriptomics analyses on two prostate cancer cell lines, LNCAP and LASCPC-01. Established from a metastatic lesion of human prostate adenocarcinoma, the LNCAP cell line is androgen-dependent and also responds to hormonal therapies. LASCPC-01 is an SCNC prostate cancer cell line with *N-Myc* gene as a driver but does not express the androgen receptor [16]. Both cell lines are well-established models to study each prostate cancer type. Comparison of cellular metabolism between two prostate cancer types provides insights into tumor pathogenesis and creates potential metabolically targeted treatments for each tumor type.

## 2. Results

### 2.1. Transcriptome and Metabolome Profiles Were Different between LASCPC-01 and LNCAP Cell Lines

**Figure 1:**
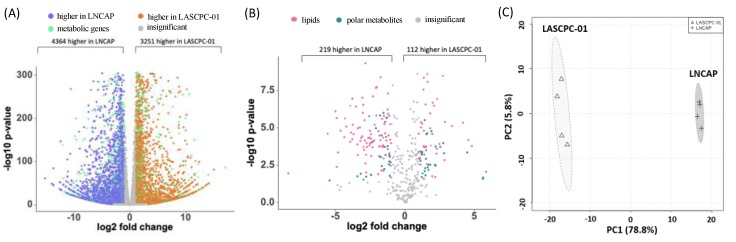
Transcriptome and metabolome profiles of LASCPC-01 and LNCAP cell lines. (**A**) 7615 transcripts were differentially expressed between LASCPC-01 and LNCAP cell lines (adjusted *p*-value < 0.05; fold change (LASCPC-01/LNCAP) > 2); Orange: expression levels were higher in LASCPC-01; Purple: expression levels were higher in LNCAP; Green: transcripts involved in metabolic processes; Grey: adjusted *p*-value > 0.05; or –2 < fold change (LASCPC-01/LNCAP) < 2. (**B**) 197 annotated metabolites were significantly different between two cell lines (adjust *p*-value < 0.05, fold change (LASCPC-01/LNCAP) > 2); Pink: lipids; Green: polar metabolites. (**C**) Principal component analysis of all annotated metabolites demonstrating the large overall metabolic differences between both cell types.

With 7615 out of 16,225 transcripts, almost half of all detected genes were differentially expressed between LASCPC-01 and LNCAP cell lines (adjusted *p*-value < 0.05, fold change > 2). The expression levels of 3251 transcripts were higher in the LASCPC-01 cell line, while 4364 transcripts were higher in the LNCAP cell line. Among 7615 significantly expressed transcripts, 1491 were involved in various metabolic processes (Figure 1A, Table S1). Using three untargeted mass spectrometry-based assays (HILIC-QExactive MS, CSH-QExactive MS, and GC-TOF MS), we identified a total of 374 metabolites in addition to detecting many unknown metabolites. More than half of all identified metabolites (197 of 374 compounds) were significantly different between two cell lines (adjusted *p*-value < 0.05, fold change > 2). Thirty-two polar metabolites and 40 lipids were higher in the LASCPC-01 cell line, while 31 polar metabolites and 94 lipids were higher in the LNCAP cell line (Figure 1B, Table S2). Principal component analysis (PCA) of 374 metabolites showed that LASCPC-01 and LNCAP cell lines were well separated on principal component 1 (PC1), explaining 78.8% of the total variance (Figure 1C), indicating a large remodeling of both lipid and polar metabolism in these prostate cancer types.

### 2.2. Gene Set Enrichment Analysis

**Figure 2:**
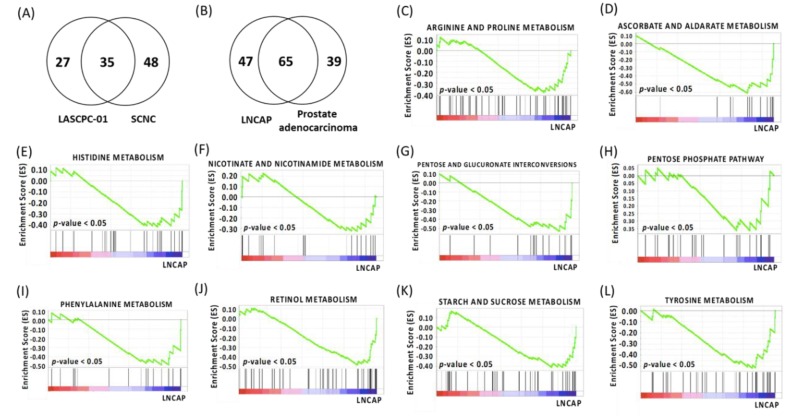
(**A**) Venn diagram of gene sets upregulated in both the LASCPC-01 cell line and patients with SCNC. (**B**) Venn diagram of gene sets upregulated in both the LNCAP cell line and patients with prostate adenocarcinoma. (**C**) Gene set enrichment (GSEA) plot depicting the enrichment of genes upregulated in arginine and proline metabolism in the LNCAP cell line (*p*-value < 0.05). (**D**) GSEA plot depicting the enrichment of genes upregulated in ascorbate and aldarate metabolism in the LNCAP cell line (*p*-value < 0.05). (**E**) GSEA plot depicting the enrichment of genes upregulated in histidine metabolism in the LNCAP cell line (*p*-value < 0.05). (**F**) GSEA plot depicting the enrichment of genes upregulated in nicotinate and nicotinamide metabolism in the LNCAP cell line (*p*-value < 0.05). (**G**) GSEA plot depicting the enrichment of genes upregulated in pentose and glucuronate interconversions pathway in the LNCAP cell line (*p*-value < 0.05). (**H**) GSEA plot depicting the enrichment of genes upregulated in pentose phosphate pathway in the LNCAP cell line (*p*-value < 0.05). (I) GSEA plot depicting the enrichment of genes upregulated in phenylalanine metabolism in the LNCAP cell line (*p*-value < 0.05). (**J**) GSEA plot depicting the enrichment of genes upregulated in retinol metabolism in the LNCAP cell line (*p*-value < 0.05). (**K**) GSEA plot depicting the enrichment of genes upregulated in starch and sucrose metabolism in the LNCAP cell line (*p*-value < 0.05). (**L**) GSEA plot depicting the enrichment of genes upregulated in tyrosine metabolism in the LNCAP cell line (*p*-value < 0.05).

While differential expression of individual metabolites (or genes) can give important clues about mechanistic aspects of cellular regulation, many compounds (and genes) are regulated in concert. In order to categorize such modules of cellular regulation, bioinformatics approaches for “gene set enrichment” (GSEA) statistics have been developed [17]. GSEA on two cell lines showed that 62 gene sets were upregulated in the LASCPC-01 cell line, among which 10 gene sets were significantly enriched at false discovery rate (FDR) < 0.25, and 27 gene sets were significantly enriched at nominal *p*-value < 0.05. A total of 112 gene sets were upregulated in the LNCAP cell line, among which 83 gene sets were significantly enriched at FDR < 0.25, and 72 gene sets were significantly enriched at nominal *p*-value < 0.05 (Table S3 and Table S4). We also performed GSEA on transcriptomics data downloaded online from 13 patients (14 biopsies) with SCNC and 74 patients (90 biopsies) with prostate adenocarcinoma [6]. The analyzed samples were metastatic biopsies from bone, lymph nodes, liver, and other soft tissues [6]. A total of 83 gene sets were upregulated in patients with SCNC, among which no gene sets were significantly enriched at FDR < 0.25, and two gene sets (cardiac muscle contraction and proximal tubule bicarbonate reclamation) were enriched at nominal *p*-value < 0.05. Ninety-four gene sets were upregulated in patients with prostate adenocarcinoma, among which no gene sets were significantly enriched at FDR < 0.25, and one gene set (riboflavin metabolism) was enriched at nominal *p*-value < 0.05. A total of 35 gene sets were upregulated in both the LASCPC-01 cell line and patients with SCNC (Figure 2A, Table S3). A total of 65 gene sets were upregulated in both the LNCAP cell line and the patients with prostate adenocarcinoma (Figure 2B, Table S4). These gene sets were involved in the metabolism of both lipids and polar metabolites. The gene sets involved in polar metabolite metabolism are summarized in Table 1 and Table 2. Significantly enriched gene sets (*p*-value < 0.05) in the LNCAP cell line are shown in Figure 2C–L.

### 2.3. Chemical Similarity Enrichment Analysis

**Figure 3:**
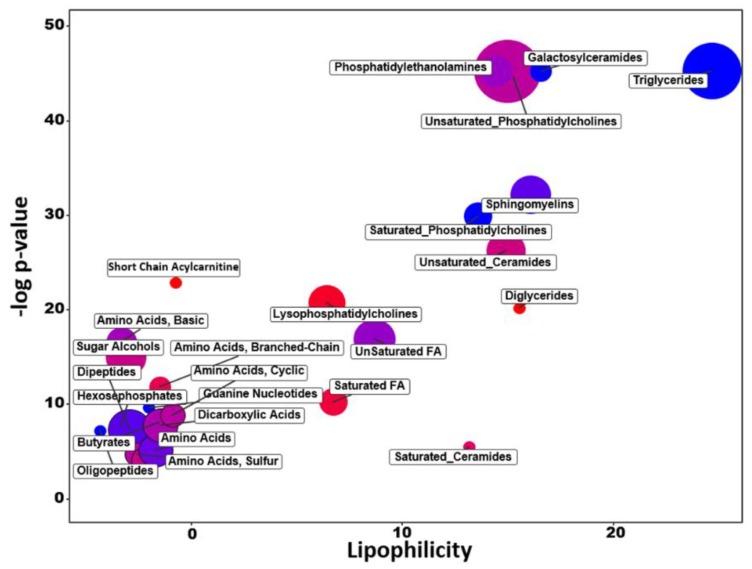
ChemRICH analysis in LASCPC-01 compared to LNCAP cells. Y-axis: significance of enriched sets of metabolites (FDR < 0.05); X-axis: average lipophilicity of sets of metabolites (cluster colors give the proportion of increased compounds in the LASCPC-01 cell line, red = increased levels in LASCPC-01 cells, blue = decreased levels in LASCPC-01); and circle size: number of detected compounds in this cluster.

Similar to GSEA, analysis of chemical similarity enrichments (ChemRICH) uses database-independent statistical models for sets of variables. ChemRICH automatically detects and labels sets of metabolites and then uses Kolmogorov–Smirnoff statistics to test the significance of differential regulation [18]. ChemRICH analysis showed that a total of 25 metabolite clusters were significantly different (FDR < 0.05) between LASCPC-01 and LNCAP cell lines (Figure 3, Table S5). For example, all compounds clustered in four of these enriched modules were found at reduced levels in LASCPC-01 cell line samples compared to LNCAP cells, specifically “triglycerides”, “galactosylceramides”, “saturated phosphatidylcholines”, and “guanine nucleotides”. In contrast, all compounds in the “diglycerides” and the “short chain acylcarnitine” clusters were found at increased levels in LASCPC-01 cells. In most other metabolite clusters, some compounds were found increased in the LASCPC-01 cell line whereas other members of these modules in the LASCPC-01 cell line were detected at reduced concentrations; such modules included “phospatidylethanolamines”, “unsaturated phosphatidylcholines”, “sphingomyelins”, “unsaturated ceramides”, “unsaturated fatty acids”, “dicarboxylic acids”, “dipeptides”, and several clusters containing a diversity of amino acids. From this statistical enrichment analysis, it became clear that lipid biosynthesis and degradation activities were different between two cell lines, indicated by a concomitant decrease in complex lipids (triglyceride, saturated phosphatidylcholine, galactosylceramide, and sphingomyelin clusters) and increase in diglycerides, lysophosphatidylcholines, and saturated fatty acids. Interestingly, differences in nitrogen metabolism is split into two parts: on the one hand, LASCPC-01 cells are enriched in branched-chain and sulfur-containing amino acids, but on the other hand, these cells were decreased in di- and oligopeptides as well as polar and acidic amino acids.

### 2.4. LASCPC-01 Exhibited a Higher Glycolytic Activity

**Figure 4:**
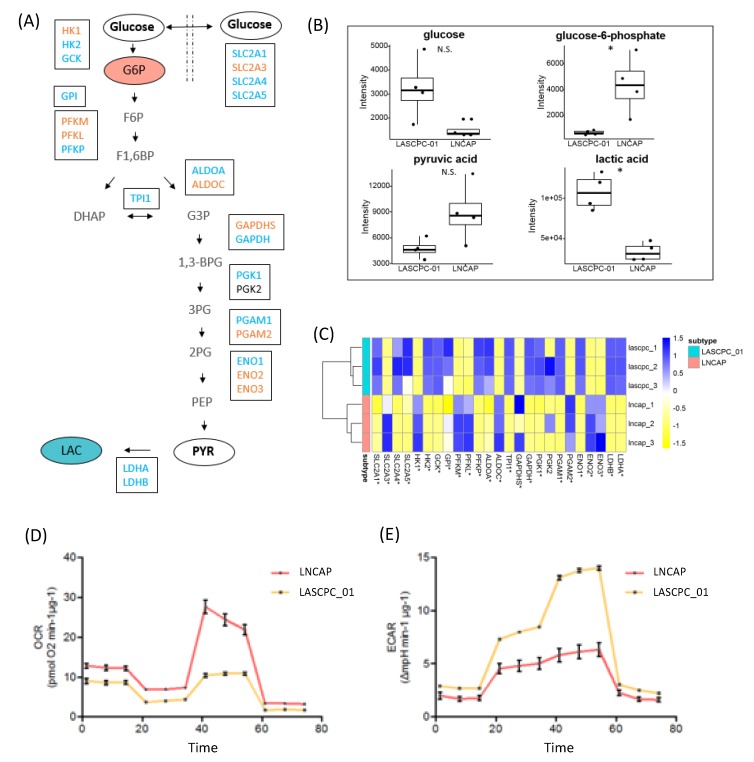
Glycolytic activity of two cell lines. (**A**) Metabolites and genes involved in the glycolysis pathway. Blue: higher in LASCPC-01; Orange: higher in LNCAP; Bold: no significant difference; Grey: not detected. (**B**) Detected metabolites involved in the glycolysis process. N.S.: no significant difference; *: *p* < 0.05. (**C**) Detected transcripts involved in glycolysis; *: *p* < 0.05. (**D**) Oxygen consumption rate of two cell lines. (**E**) Extracellular acidification rate of two cell lines.

Although metabolomics showed no significant difference in the level of pyruvate between LASCPC-01 and LNCAP cell lines, GSEA revealed the upregulation of pyruvate metabolism in both the LASCPC-01 cell line and in patients with SCNC (Table 1). We further checked other glycolysis metabolites and found that a lower level of glucose-6-phosphate but a higher level of lactate was observed in LASCAP-01 cells compared to LNCAP cells (Figure 4A,B). Interestingly, the expression levels of two lactate dehydrogenase (LDH) isoforms, LDHA and LDHB, both were higher in LASCPC-01 cells (Figure 4C), consistent with the higher level of lactate observed in the LASCPC-01 cell line. The seahorse assay also showed that the LASCPC-01 cell line exhibited a higher glycolytic activity with a lower oxygen consumption rate (Figure 4D) and a higher extracellular acidification rate (Figure 4E). Combined, these data showed that LASCAP-01 cells showed a stronger Warburg effect of aerobic glycolysis.

### 2.5. Elevated Levels of Serine and Glycine in LNCAP

**Figure 5:**
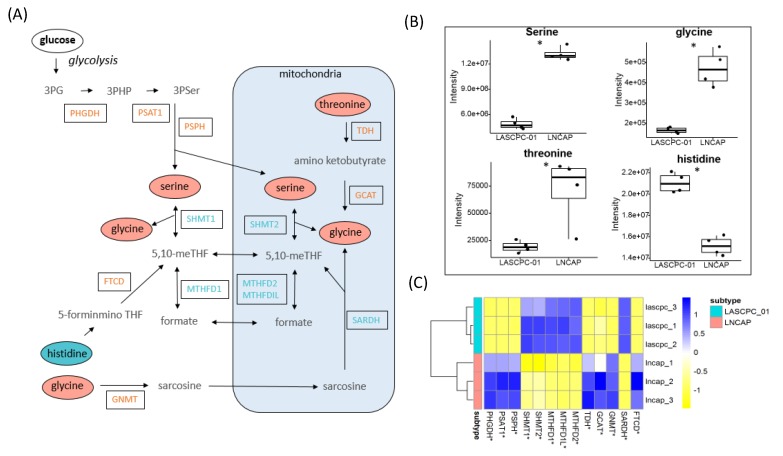
Serine and glycine metabolism of two cell lines. (**A**) Metabolites and genes involved in serine and glycine metabolism. Blue: higher in LASCPC-01; Orange: higher in LNCAP; Bold: no significant difference; Grey: not detected. (**B**) Detected metabolites involved in serine and glycine metabolism; *: *p* < 0.05. (**C**) Detected transcripts involved in the serine and glycine metabolism; *: *p* < 0.05.

GSEA showed that glycine, serine, and threonine metabolism was upregulated in both the LNCAP cell line and in patients with prostate adenocarcinoma (Table 2). Metabolomics data showed that the levels of serine, glycine, and threonine were higher in the LNCAP cell line (Figure 5A,B). Expression levels of genes involved in serine biosynthesis were also higher in the LNCAP cell line, including phosphoglycerate dehydrogenase (PHGDH), phosphoserine aminotransferase 1 (PSAT1), and phosphoserine phosphatase (PSPH) (Figure 5A,C). The expression levels of genes involved in 5,10-methylenetetrahydrofolate (5,10-meTHF) and formate biosynthesis, however, were lower in LNCAP cells, including serine hydroxymethyltransferase (SHMT1 and SHMT2) and methylenetetrahydrofolate dehydrogenase (MTHFD1, MTHFD2, and MTHFD1L) (Figure 5A,C). In contrast to the higher level of serine and glycine, the overall histidine level was lower in LNCAP cells, potentially because of higher expression of formimidoyltransferase-cyclodeaminase (FTCD), which is involved in histidine degradation and 5,10-meTHF formation (Figure 5A–C). Correlating with serine concentrations, the level of threonine was higher in LNCAP cells, as well as the expression levels of genes involved in the conversion of threonine to glycine, threonine dehydrogenase (TDH), and glycine C-acetyltransferase (GCAT) (Figure 5A–C). In combination, these data supported the hypothesis that LNCAP lines diverted metabolic flux away from glycolysis, using the glycolytic intermediate 3-phosphoglycerate as substrate for increased biosynthesis of serine. Serine/glycine transformations charge one-carbon metabolism, which is involved in many reactions, notably also in nucleotide biosynthesis.

### 2.6. Citrate Accumulated in LNCAP Cells

**Figure 6:**
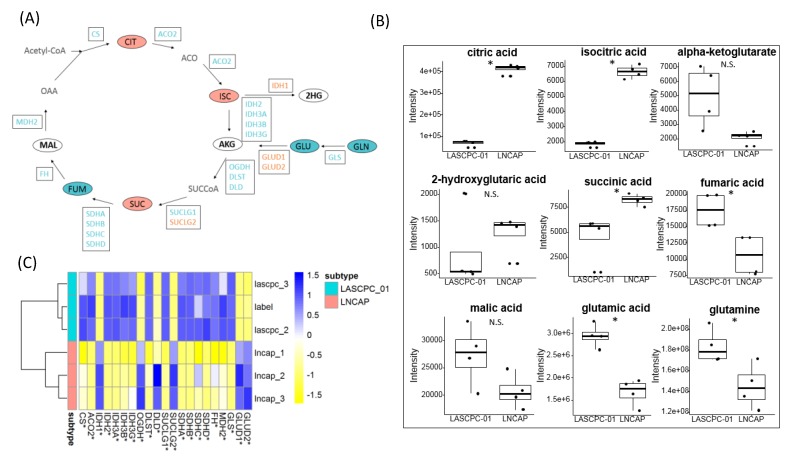
Tricarboxylic Acid (TCA) cycle and citrate production of two cell lines. (**A**) Metabolites and genes involved in the TCA cycle; Blue: higher in LASCPC-01; Orange: higher in LNCAP; Bold: no significant difference; Grey: not detected. (**B**) Detected metabolites involved in the TCA cycle; *: *p* < 0.05; N.S.: no significant difference. (**C**) Detected transcripts involved in TCA cycle; *: *p* < 0.05.

Higher levels of citrate, isocitrate, and succinate and lower levels of fumarate, glutamate, and glutamine were found in LNCAP cells, but there was no significant difference in the levels of alpha-ketoglutarate, malate, and 2-hydroxyglutarate (Figure 6A,B). The expression levels of genes encoding aconitase 2 (ACO2) and most enzymes in the tricarboxylic acid (TCA) cycle were higher in LASCPC-01 cells, except isocitrate dehydrogenase 1 (IDH1) and succinate-CoA ligase GDP-forming beta subunit (SUCLG2), which were higher in LNCAP cells (Figure 6A,C). In addition, the expression levels of glutamate dehydrogenase (GLUD1 and GLUD2) were higher in LNCAP cells, catalyzing the oxidative deamination of glutamate to alpha-ketoglutarate (Figure 6A,C). Combined, these data showed that citrate oxidation or mitochondrial oxidative phosphorylation activity was different between two cell lines. In LNCAP cells, citrate accumulated, which could be exported out of the mitochondria into the cytosol and used for lipogenesis.

### 2.7. Short-Chain Acylcarnitines Were Lower in LNCAP

**Figure 7:**
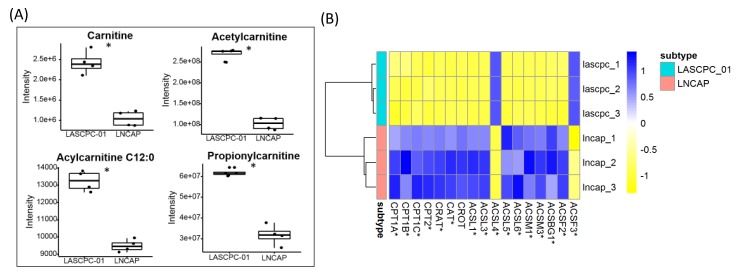
Carnitine system in two cell lines. (**A**) Lower levels of carnitine metabolites were found in LNCAP cell lines. (**B**) Detected transcripts in the carnitine transport system; *: *p* < 0.05.

Lower levels of carnitine, the inner membrane transporter molecule of mitochondrial lipid metabolism, was observed in LNCAP cells, along with lower levels of short-chain acylcarnitines C2:0, C3:0, and C12:0 (Figure 7A). Conversely, gene expression for biosynthesis and use of acylcarnitines was activated, including genes encoding carnitine O-palmitoyltransferase 1 (CPT1A, CPT1B, and CPT1C), carnitine O-palmitoyltransferase 2 (CPT2), carnitine O-acetyltransferase (CRAT), catalase (CAT), and carnitine O-octanoyltransferase (CROT) (Figure 7B). Expression levels of many members in the acyl-coenzyme A synthetase family were also higher in LNCAP (Figure 7B). As the carnitine system plays a key role in fatty acyl moiety transport and fatty acid metabolism, the reduced levels of carnitine and short-chain acylcarnitines in the LNCAP cell line might lead to reduced fatty acid oxidation activity.

### 2.8. Different Lipid Metabolism

**Figure 8:**
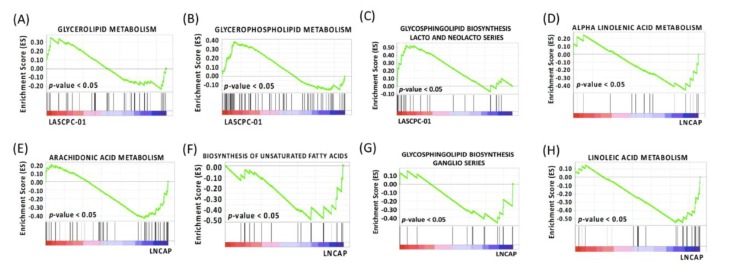
(**A**) GSEA plot depicting the enrichment of genes upregulated in glycerolipid metabolism in the LASCPC-01 cell line (*p*-value < 0.05). (**B**) GSEA plot depicting the enrichment of genes upregulated in glycerophospholipid metabolism in the LASCPC-01 cell line (*p*-value < 0.05). (**C**) GSEA plot depicting the enrichment of genes upregulated in glycosphingolipid biosynthesis lacto and neolacto series in the LASCPC-01 cell line (*p*-value < 0.05). (**D**) GSEA plot depicting the enrichment of genes upregulated in alpha linolenic acid metabolism in the LNCAP cell line (*p*-value < 0.05). (**E**) GSEA plot depicting the enrichment of genes upregulated in arachidonic acid metabolism in the LNCAP cell line (*p*-value < 0.05). (**F**) GSEA plot depicting the enrichment of genes upregulated in biosynthesis of unsaturated fatty acids in the LNCAP cell line (*p*-value < 0.05). (**G**) GSEA plot depicting the enrichment of genes upregulated in glycophingolipid biosynthesis ganglio series in the LNCAP cell line (*p*-value < 0.05). (**H**) GSEA plot depicting the enrichment of genes upregulated in linoleic acid metabolism in the LNCAP cell line (*p*-value < 0.05).

Lipidomics data revealed a profound difference between LASCPC-01 and LNCAP cell lines, as shown by the ChemRICH plot (Figure 3). In addition, GSEA showed that four gene sets were upregulated in both the LASCPC-01 cell line and patients with SCNC, while eight gene sets were upregulated in both the LNCAP cell line and in patients with prostate adenocarcinoma (Table 3 and Table 4). Significantly enriched gene sets (*p*-value < 0.05) in LASCPC-01 and LNCAP are shown in Figure 8A–C and Figure 8D–H, respectively. Altogether, these data suggested lipid metabolism was different in two prostate cancer cell lines.

## 3. Discussion

Cancer cells reprogram their metabolism to supply energetic and biosynthetic demands and maintain viability and proliferation [7]. Tumor-associated metabolic alterations are involved in all stages of cell–metabolite interactions [7]. Some metabolic alterations in prostate cancer have been previously reported, for example, the increase of aerobic glycolysis in advanced diseases or dysregulation of lipid metabolism [9,19,20,21,22]. However, most studies only focused on specific types of prostate cancer and the heterogeneity represented by the Gleason gradings, despite the heterogeneity of different types of prostate cancers. We here show for the first time that transcriptome and metabolome profiles were different between prostate adenocarcinoma (LNCAP) and prostate small cell neuroendocrine carcinoma (LASCPC-01) cells, providing necessary information to discover novel therapeutic targets and treatment strategies for these different types of prostate cancer.

A special phenotype of the prostate gland is the accumulation and secretion of high levels of citrate. In the mitochondria of most mammalian cells, citrate is oxidized through the TCA cycle. However, in benign prostate cells, zinc accumulates and inhibits the activity of mitochondrial aconitase [23] that catalyzes the stereospecific isomerization of citrate to isocitrate. While this TCA cycle enzyme usually does not have a regulatory role, in prostate cells the Zn-mediated inhibition of aconitase leaves citrate as the final product of glucose metabolism. In malignant prostate cells, however, citrate is oxidized in the TCA cycle, and the resulting mitochondrial NADH is converted to ATP [15]. Lower levels of citrate and higher expression levels of the gene encoding aconitase 2 in LASCPC-01 cells (Figure 6A–C) supported the concept of energetically efficient SCNC through reactivation of aconitase and subsequent citrate oxidation. A reduced level of citrate is featured in the more aggressive prostate phenotype SCNC. The citrate metabolic pathway can be a potential therapeutic target for treatment of SCNC, which needs further investigation.

To ensure that prostate cancer cells have the required energy for rapid proliferation, maintaining the availability of acetyl-CoA is necessary [13]. Alterations in fatty acid metabolism have been suggested to provide acetyl-CoA and ATP [13]. Citrate accumulated in LNCAP cells (Figure 6A,B). Citrate is exported out of the mitochondria into the cytosol and subsequently cleaved by ATP citrate lyase (ACLY) to oxaloacetate and acetyl-CoA, which is used for lipogenesis. Higher expression levels of ACLY and fatty acid synthase (FASN) in the LNCAP cell line (Table S1) support this hypothesis. Meanwhile, the reduced levels of carnitine and short-chain acylcarnitines in the LNCAP cell line may reduce fatty acid oxidation activity (Figure 7A). In combination, both mechanisms may lead to the accumulation of triglycerides observed in LNCAP cells (Figure 2). 

The carnitine transport system is essential for transport of fatty acyl moieties from the cytosol to mitochondria and vice versa. This transport system has been considered as a gridlock to control metabolic flexibility of cancer cells [24]. As a pivotal mediator, the carnitine system tunes the switch between glucose and fatty acid metabolism [24]. Here, we found decreased levels of carnitine, and short chain acylcarnitines in LNCAP cells (Figure 7A). Potentially in response to the lower levels of carnitine and acylcarnitines, gene expressions in the carnitine system were activated, including genes encoding the main transport proteins CPT1A, CPT1B, CPT1C, CPT2, and CRAT (Figure 7B). Located on the outer membrane of mitochondria, CPT1 converts long-chain fatty acid-CoAs into acylcarnitine derivatives. It has been reported the blockage of CPT1A decreased the viability of LNCAP and other androgen-dependent prostate cell lines [25]. CPT2, which is located in the inner mitochondria membrane, reconverts long-chain acylcarnitines to their acyl-CoA counterparts for the use of subsequent beta-oxidation and acetyl-CoA production. CRAT converts short-chain acetyl-CoAs to respective acetylcarnitne, which could be exported to the cytoplasm. The carnitine transport system is essential for cancer cells to gain energy from beta-oxidation of lipids. Regulation of carriers and enzymes that modulate β-oxidation is of extreme interest in cancer cells [24]. Tumorigenic potential may be affected significantly due to disruption of the carnitine transport system even in the presence of a compensatory metabolic pathway. The deficiency of carnitines in the LNCAP cell line may lead to reduction in fatty acid oxidation and further accumulation of triglycerides. On the other hand, elevated levels of carnitines in the more lethal and aggressive SCNC greatly increased fatty acid oxidation to provide energy for tumor growth. Targeting the carnitine system to control the energy source for SCNC is a striking discovery for future prostate cancer research.

The importance of serine/glycine metabolism for one-carbon metabolic transformations has been well documented in cancer biology [26,27,28]. Serine is involved in cancer metabolism through amino acid transport, nucleotide synthesis, redox homeostasis, and folate metabolism [29]. Serine is the central node for the biosynthesis of a range of metabolites. For instance, serine/glycine conversion supplies carbon to the one-carbon pool, which is essential for DNA methylation and de novo nucleotide biosynthesis [30]. A key enzyme in one-carbon metabolism is SHMT, as it is involved in two pathways for chemotherapeutic intervention, serine–glycine metabolism, and nucleotide biosynthesis [29]. Two SHMT encoding genes are found in the human genome, SHMT1 encoding the cytosolic form and SHMT2 encoding the mitochondrial form, both of which are transcriptional targets of oncogene *c-Myc* [31]. In this study, higher expression levels of genes involved in serine biosynthesis were observed in LNCAP cells as well as elevated levels of glycine and threonine (Figure 5A–C). In LASCPC-01 cells, higher expression levels of genes encoding SHMT1 and SHMT2 and decreased availability of serine and glycine has been observed, indicating a higher demand of serine and glycine for multiple purposes in this aggressive subtype of prostate cancer.

One limitation of our study is that the number of samples is low. However, this study provides the proof of concept for integration of multi-omics, including transcriptomics, metabolomics, and lipidomics, to compare the metabolic differences in the prostate SCNC cell line LASCPC-01 and the prostate adenocarcinoma cell line LNCAP. The LNCAP cell line showed elevated levels of serine and glycine, while LASCPC-01 exhibited a higher glycolytic activity, an increased level of carnitines, and decreased levels of citrate and triglycerides. Compared to prostate adenocarcinoma, the more aggressive SCNC requires more energy, which is generated from aerobic glycolysis, oxidative phosphorylation, and carnitine-mediated fatty acid beta-oxidation, to meet the needs for rapid tumor growth and proliferation in the microenvironment. Future research targeting these metabolic pathways to restrict the energy supply for SCNC tumor cells may lead to new treatment therapies for this highly aggressive prostate cancer.

## 4. Materials and Methods 

### 4.1. Materials

LNCAP cells were purchased from American Type Culture Collection (ATCC, Manassas, VA, USA) and grown in RPMI1640 media supplemented with 10% fetal bovine serum (FBS) (Atlanta Biologicals, Flowery Branch, GA, USA). LASCPC-01 cells, a kind gift from Dr. Owen Witte’s laboratory (University of California, Los Angeles, CA, USA), were established by transducing benign human prostate epithelial cells to stably express myristoylated *AKT1* and *N-Myc* [16]. LASCPC-01 cells were grown in HITES media (RPMI with 10 nM hydrocortisone, 10 nM beta-estradiol (Sigma), insulin-transferrin-selenium, and Glutamax (Life Technologies, Carlsbad, CA, USA)) supplemented with 10% FBS.

### 4.2. Transcriptomics Library Construction and Data Analysis

Total RNA was extracted with Trizol/CHCl3 (Life Technologies, Carlsbad, CA, USA) according to the manufacturer’s protocol, and the aqueous phase was put through the Qiagen RNEasy kit for cleanup. Samples for RNA-sequencing were prepared using the Agilent SureSelect Strand-Specific mRNA Library Preparation Protocol (Version A.2, September 2013, Agilent Technologies, Santa Clara, CA, USA). Briefly, poly-A RNA was purified from 1 µg total RNA per sample using oligo dT magnetic beads and was chemically fragmented. Next, first-strand cDNA was synthesized using the First Strand Master Mix (Agilent) followed by purification using AMPure XP beads. Next, second-strand cDNA was synthesized and the 3’ ends of second-strand cDNA were adenylated, followed by adapter ligation and AMPure XP bead purification. Ligated DNA was PCR amplified for 14 cycles followed by purification with AMPure XP beads. Quality of the resulting libraries was assessed with an Agilent 2100 Bioanalyzer DNA 1000 Assay. Libraries were sequenced on an Illumina (San Diego, CA, USA) HiSeq as single-end 50 bp reads. Three biological replicate RNA samples from each cell line were used for sequencing. Transcriptomics data analysis was performed using the Tuxedo Suite [32]. Each sample was mapped independently to the human genome build GRCh37/hg19 using Tophat version 2.0.9. Transcript assembly and quantification was done with Cufflinks version 2.1.1. The expression levels were normalized by Cuffnorm function in Cufflinks version 2.1.1 and reported in fragments per kilobase of transcript per million mapped reads (FPKM) units. Differential gene expression analysis was done with Cuffdiff function in Cufflinks version 2.1.1.

### 4.3. Public Transcriptomics Data

We downloaded publicly available transcriptomics data from a multi-institutional prospective study, which contained 14 biopsies from 13 patients with SCNC and 90 biopsies from 74 patients with prostate adenocarcinoma [6]. The analyzed samples were metastatic biopsies from bone, lymph nodes, liver, and other soft tissues.

### 4.4. Cellular Respiration

Oxygen consumption and extracellular acidification rates were carried out in an XF96 Seahorse Analyzer (Agilent/Seahorse Bioscience, Billerica, MA, USA). Cells were plated in the wells of 96-well plates (8 × 10^3^ cells/well; XF96 plates, Seahorse Bioscience, North Billerica, MA, USA) and incubated at 37 °C overnight. The next day, cells were treated with indicated drugs for 24 h, and then the medium was changed to XF Assay Medium and loaded with glucose, oligomycin, and 2-DG, respectively, following the manufacturer’s recommendation. Similarly, Mito Fuel Flex Tests were performed on an XFe96 Bioanalyzer. At 24 h post-treatment, all assays were performed following the manufacturer’s protocols.

### 4.5. Profiling Primary Metabolism

Primary metabolites were analyzed by gas chromatography-time of flight mass spectrometry (GC-TOF MS). Four biological replicates from different cryovials for each cell line (2 million cells per sample) were homogenized using a GenoGrinder 2010 (SPEX SamplePrep, Metuchen, NJ, USA) for 30 s at 1500 rpm and centrifuged at 14,000× *g* for 2 min. Cells were not washed before the homogenization step to avoid metabolic perturbation during the wash step [33]. Cells were extracted with 1 mL of −20 °C cold, degassed acetonitrile:isopropanol:water (3:3:2, *v*/*v*/*v*). Supernatant (500 μL) was evaporated to dryness using a CentriVap (Labconco, Kansas, MO, USA). Metabolites were derivatized in two steps, as published previously [34]: first, carbonyl groups were protected by methoximation; second, acidic protons were exchanged against trimethylsilyl-groups to increase volatility. A 0.5 μL sample was injected with 25 s splitless time on an Agilent 6890 GC (Agilent Technologies, Santa Clara, CA, USA) using a Restek Rtx-5Sil MS column (30 m × 0.25 mm × 0.25 μm) with 10 m guard column (10 m × 0.25 mm × 0.25 μm) and 1 mL/min helium gas flow. Oven temperature was held at 50 °C for 1 min and then ramped up to 330 °C at 20 °C/min and held for 5 min. Data were acquired at −70 eV electron ionization at 17 spectra/s from 85 to 500 Da at 1850 V detector voltage on a Leco Pegasus IV time-of-flight mass spectrometer (Leco Corporation, St. Joseph, MI, USA). The transfer line temperature was held at 280 °C with an ion source temperature set at 250 °C. Standard metabolite mixtures and blank samples were injected at the beginning of the run and every ten samples throughout the run for quality control. Raw data were preprocessed by ChromaTOF version 4.50 for baseline subtraction, deconvolution, and peak detection. Specifically, 3 s peak width, baseline subtraction just above the noise level, automatic mass spectral deconvolution, and peak detection at signal/noise levels of 5:1 throughout the chromatogram were used. Binbase version 5.0.3 was used for metabolite annotation and reporting [35,36]. The following settings were used by the Binbase algorithm (rtx5): validity of chromatogram 10^7^ counts/s, unbiased retention index marker detection, MS similarity >800, retention index calculation by 5th order polynomial regression, retention index window 2000 units, and validation of unique ions and apex masses.

### 4.6. Profiling Biogenic Amines

Hydrophilic interaction liquid chromatography (HILIC) with quadrupole orbital ion trap high field mass spectrometry (Q-Exactive HF MS) was used for the analysis of biogenic amine, dipeptides, methylated metabolites, and other polar metabolites. Four biological replicates from different cryovials for each cell line (2 million cells per sample) were homogenized, as given above, and extracted with 225 μL of −20 °C cold, degassed methanol and 750 μL of methyl tertiary butyl ether (MTBE, Sigma Aldrich, St. Louis, MO, USA). Methanol contained the following internal standards for quality control and retention time correction: ceramide (d18:1/17:0), cholesterol-d7, DG (12:0/12:0/0:0), DG (18:1/2:0/0:0), MG (17:0/0:0/0:0), LPC (17:0), LPE (17:1), palmitic acid-d3, PC (17:0/0:0), PC (12:0/13:0), PE (17:0/17:0), PE (17:1/0:0), PG (17:0/17:0), SM (d18:0/17:0), sphingosine C17, TG (17:0/17:1/17:0)-d5. MTBE contained cholesteryl ester 22:1 as an internal standard. Phase separation was induced by adding 188 μL of liquid chromatography–mass spectrometry (LC-MS) grade water, followed by centrifugation at 14,000× *g* for 2 min. The upper non-polar phase (350 μL) and bottom polar phase (125 μL) were collected separately and evaporated to dryness. The polar layer was re-suspended in 60 μL 4:1 acetonitrile and water (*v*/*v*) with internal standards. Samples were then vortexed, sonicated for 5 min, and centrifuged for 2 min at 14,000× *g*. A 5 μL re-suspended sample was injected using a Vanquish UHPLC system (Thermo Scientific, Waltham, MA, USA) onto a Acquity UPLC BEH Amide column (150 mm × 2.1 mm × 1.7 μm) coupled to an Acquity VanGuard BEH Amide pre-column (5 mm × 2.1 mm × 1.7 μm, Waters, Milford, MA, USA). The column was maintained at 45 °C. LC-MS grade water (100%) with 10 mM ammonium formate and 0.125% formic acid (Sigma–Aldrich, St. Louis, MO, USA) was used as mobile phase A and 95:5 *v*/*v* acetonitrile:water (*v*/*v*) with 10 mM ammonium formate and 0.125% formic acid (Sigma–Aldrich, St. Louis, MO, USA) was used as mobile phase B. The gradient was performed as follows: 0–2 min 100% B, 7.7 min 70% B, 9.5 min 40% B, 10.25 min 30% B, 12.75 min 100% B, and then isocratic until 16.75 min with a flow rate of 0.4 mL/min. A ThermoFisher Q-Exactive HF with a HESI-II ion source (Thermo Scientific, Waltham, MA, USA) was used to collect spectra with a data-dependent MS/MS spectra acquisition method in ESI positive mode. Other parameters were as follows: spray voltage, 3.6 kV; capillary temperature, 300 °C; aux gas heater temperature, 370 °C; aux gas flow, 25 arbitrary units; sweep gas flow, 2 arbitrary units; sheath gas pressure, 60 psi; MS1 mass range *m*/*z* 60–900; MS1 resolving power, 60,000 FWHM (*m*/*z* 200); number of data-dependent scans per cycle, four; MS2 resolving power, 15,000 FWHM (*m*/*z* 200); acquisition speed: 2 MS1 spectra/s; and normalized collision energy, 20%, 30%, and 40%. Method blank and human plasma (BioIVT, Westbury, NY, USA) samples were injected at the beginning of the run and every ten samples throughout the run as quality control samples.

### 4.7. Profiling Complex Lipids

The dried non-polar phase extract was re-suspended in 9:1 methanol:toluene (*v*/*v*) containing 50 ng/mL 12-[[(cyclohexylamino)carbonyl]amino]-dodecanoic acid (CUDA, Cayman Chemical, Ann Arbor, MI, USA) as an internal standard. A 5 μL re-suspended sample was injected using a Vanquish UHPLC system (Thermo Scientific, Waltham, MA, USA) onto an Acquity UPLC CSH C18 (100 mm × 2.1 mm × 1.7 μm) column coupled to an Acquity VanGuard CSH C18 pre-column (5 mm × 2.1 mm × 1.7 μm, Waters, Milford, MA, USA). The column was maintained at 65 °C throughout the run. Mobile phase A consisted of 60:40 acetonitrile:water (*v*/*v*). Mobile phase B consisted of 90:10 isopropanol:acetonitrile (*v*/*v*). Ammonium formate (10 mM) and 0.1% formic acid (Sigma–Aldrich, St. Louis, MO, USA) were used as modifiers for the positive mode. Ammonium acetate (10 mM) (Sigma–Aldrich, St. Louis, MO) was used as modifier for the negative mode. The gradient was 0 min 15% B, 0–2 min 30% B, 2–2.5 min 48% B, 2.5–11 min 82% B, 11–11.5 min 99% B, 11.5–12 min 99% B, 12–12.1 min 15% B, and 12.1-15 min 15% B. The flow rate was 0.6 mL/min. A Thermo Fisher Q-Exactive HF mass spectrometer with a HESI-II ion source (Thermo Scientific, Waltham, MA, USA) was used to collect spectra with data-dependent MS/MS spectra acquisition. Scan range was from 50 to 1700 Da. Spectra were collected in both ESI(+) and ESI(−) mode. Simultaneous MS1 and MS/MS (data-dependent MS/MS) acquisition was used. The parameters were: spray voltage, ±3.6 kV; capillary temperature, 300 °C; aux gas heater temperature, 370 °C; aux gas flow, 25 arbitrary units; sheath gas pressure, 60 arbitrary units; sweep gas flow, 2 arbitrary units; MS1 mass range, *m*/*z* 220–1700; MS1 resolving power, 120,000 FWHM (*m*/*z* 200); number of data-dependent scans per cycle, three; MS/MS resolving power, 15,000 FWHM (*m*/*z* 200); MS1 acquisition speed, 2 spectra/s; normalized collision energy, 20%, 30%, and 40%. Method blank and human plasma (BioIVT, Westbury, NY, USA) were injected at the beginning of the run and every ten samples throughout the run as quality control samples.

### 4.8. Liquid chromatography–mass spectrometry (LC-MS) Data Processing

LC-MS raw data files were converted to ABF format using ABF converter (https://www.reifycs.com/AbfConverter/). MS-DIAL version 2.94 was used for peak picking, alignment, deconvolution, and identification [37]. The parameters used in MS-DIAL were as follows: smoothing method, linear-weighted moving average; smoothing level, three scans; minimum peak width, five scans; minimum peak height, 50,000; mass slice width, 0.1 Da; and sigma window value for deconvolution, 0.01. MS-FLO was used to identify ion adducts, duplicate peaks, and isotopic features [38]. For both lipidomics and HILIC datasets, retention time - *m*/*z* libraries and MS/MS spectra databases were used for compound identification, as uploaded to MassBank of North America. Features that were present in at least 50% of samples in each cell line were reported.

### 4.9. Statistics 

To scale each sample, data were normalized using the sum of all identified metabolites. The Mann–Whitney U test was performed on both metabolomics and lipidomics datasets with the Benjamini–Hochberg procedure to control the false discovery rate (FDR) [39]. Principal component analysis (PCA) plots were generated using MetaboAnalyst 4.0 [40]. Chemical similarity set enrichment analysis was performed using ChemRICH software (updated on Nov. 2017) [18]. Gene set enrichment analysis was performed using GSEA software version 3.0 with Molecular Signature Database (MSigDB) collection 2, KEGG pathway database version 6.2 [17]. Phenotype permutations were performed on both cell lines and human tissue gene expression data with a permutation number of 1000. A weighted enrichment statistic was used. Volcano plots were generated with R (version 3.5.1) ggplot2 package. Heatmaps were generated with R (version 3.5.1) pheatmap package using Euclidean clustering distances.

## Figures and Tables

**Table 1:** Upregulated gene sets involved in the metabolism of polar metabolites in the LASCPC-01 cell line and SCNC patients.

Gene Sets	LASCPC-01				SCNC			
	Number	ES	*p-*value	FDR	Number	ES	*p*-value	FDR
Glyoxylate and Dicarboxylate Metabolism	7 (16)	0.45	0.08	0.31	4 (16)	0.21	0.93	0.99
Cysteine and Methionine Metabolism	17 (30)	0.42	0.08	0.28	10 (34)	0.23	0.82	1.00
Pyruvate Metabolism	8 (36)	0.33	0.16	0.31	8 (39)	0.30	0.50	1.00
Pyrimidine Metabolism	40 (94)	0.31	0.19	0.30	16 (95)	0.20	0.77	1.00
Inositol Phosphate Metabolism	13 (53)	0.26	0.20	0.28	3 (54)	0.14	0.99	0.98
Nitrogen Metabolism	7 (23)	0.38	0.20	0.31	9 (23)	0.42	0.34	1.00
Galactose Metabolism	7 (24)	0.33	0.30	0.33	7 (26)	0.29	0.65	1.00
One Carbon Pool By Folate	4 (17)	0.29	0.49	0.47	5 (17)	0.28	0.73	1.00

Note: Number: number of genes contributing to the leading-edge subset within the gene set (number of genes in the gene set after filtering out these genes not in the expression dataset); ES: enrichment score; *p*-value: nominal *p*-value not adjusted for gene set size or multiple hypothesis testing; and FDR: false discovery rate.

**Table 2:** Upregulated gene sets involved in the metabolism of polar metabolites in the LNCAP cell line and prostate adenocarcinoma patients.

Gene Sets	LNCAP				Prostate Adenocarcinoma			
	Number	ES	*p*-value	FDR	Number	ES	*p*-value	FDR
Arginine and Proline Metabolism	17 (49)	−0.38	0	0.11	14 (52)	−0.31	0.53	0.94
Ascorbate and Aldarate Metabolism	11 (18)	−0.61	0	0.18	8 (16)	−0.61	0.21	1.00
Histidine Metabolism	12 (27)	−0.42	0	0.13	5 (28)	−0.24	0.88	0.97
Nicotinate and Nicotinamide Metabolism	8 (21)	−0.33	0	0.15	7 (22)	−0.28	0.83	0.99
Pentose and Glucuronate Interconversions	7 (20)	−0.54	0	0.17	8 (18)	−0.63	0.14	1.00
Pentose Phosphate Pathway	9 (25)	−0.36	0	0.10	18 (26)	−0.20	0.97	0.98
Phenylalanine Metabolism	10 (18)	−0.49	0	0.12	6 (18)	−0.40	0.50	0.99
Retinol Metabolism	17 (48)	−0.48	0	0.10	20 (54)	−0.42	0.59	0.98
Starch and Sucrose Metabolism	13 (41)	−0.41	0	0.10	8 (43)	−0.38	0.47	0.97
Tyrosine Metabolism	14 (38)	−0.52	0	0.10	13 (42)	−0.40	0.40	0.99
Butanoate Metabolism	10 (30)	−0.41	0.09	0.13	8 (34)	−0.39	0.29	0.95
Glycine Serine and Threonine Metabolism	12 (29)	−0.40	0.10	0.18	4 (31)	−0.31	0.76	1.00
Tryptophan Metabolism	15 (37)	−0.34	0.10	0.30	8 (39)	−0.27	0.81	0.97
Valine Leucine and Isoleucine Degradation	20 (44)	−0.31	0.30	0.33	12 (44)	−0.35	0.31	0.99
Amino Sugar and Nucleotide Sugar Metabolism	14 (43)	−0.22	0.31	0.42	18 (44)	−0.33	0.40	0.99

Note: Number: number of genes contributing to the leading-edge subset within the gene set (number of genes in the gene set after filtering out these genes not in the expression dataset); ES: enrichment score; *p*-value: nominal *p*-value not adjusted for gene set size or multiple hypothesis testing; a *p*-value of zero (0) indicates an actual *p*-value of less than 0.001; and FDR: false discovery rate.

**Table 3:** Upregulated gene sets in lipid metabolism in the LASCPC-01 cell line and SCNC patients.

Gene Sets	LASCPC-01				SCNC			
	Number	ES	*p*-value	FDR	Number	ES	*p*-value	FDR
Glycerolipid Metabolism	7 (39)	0.34	0	0.30	5 (43)	0.27	0.68	1.00
Glycerophospholipid Metabolism	20 (67)	0.38	0	0.31	9 (70)	0.25	0.73	1.00
Glycosphingolipid Biosynthesis Lacto and Neolacto Series	9 (24)	0.53	0	0.19	11 (25)	0.39	0.41	1.00
Ether Lipid Metabolism	7 (26)	0.31	0.19	0.30	9 (29)	0.37	0.39	1.00

Note: Number: number of genes contributing to the leading-edge subset within the gene set (number of genes in the gene set after filtering out these genes not in the expression dataset); ES: enrichment score; *p*-value: nominal *p*-value not adjusted for gene set size or multiple hypothesis testing; a *p*-value of zero (0) indicates an actual *p*-value of less than 0.001; and FDR: false discovery rate.

**Table 4:** Upregulated gene sets in lipid metabolism in the LNCAP cell line and prostate adenocarcinoma patients.

Gene Sets	LNCAP				Prostate Adenocarcinoma			
	Number	ES	*p*-value	FDR	Number	ES	*p*-value	FDR
Alpha Linolenic Acid Metabolism	5 (15)	−0.46	0	0.13	6 (18)	−0.34	0.70	0.96
Arachidonic Acid Metabolism	21 (47)	−0.43	0	0.10	24 (57)	−0.37	0.43	0.96
Biosynthesis of Unsaturated Fatty Acids	11 (20)	−0.48	0	0.10	2 (20)	−0.27	0.77	0.98
Glycosphingolipid Biosynthesis Ganglio Series	6 (15)	−0.47	0	0.25	9 (15)	−0.31	0.77	1.00
Linoleic Acid Metabolism	14 (25)	−0.54	0	0.10	8 (28)	−0.32	0.79	0.96
Glycosylphosphatidylinositol Gpi Anchor Biosynthesis	12 (24)	−0.26	0.29	0.46	13 (25)	−0.34	0.36	0.93
Fatty Acid Metabolism	18 (39)	−0.31	0.29	0.19	16 (42)	−0.45	0.20	1.00
Sphingolipid Metabolism	5 (33)	−0.25	0.48	0.51	10 (36)	−0.32	0.39	0.98

Note: Number: number of genes contributing to the leading-edge subset within the gene set (number of genes in the gene set after filtering out these genes not in the expression dataset); ES: enrichment score; *p*-value: nominal *p*-value not adjusted for gene set size or multiple hypothesis testing; a *p*-value of zero (0) indicates an actual *p*-value of less than 0.001; and FDR: false discovery rate.

## Supplementary Materials

The following are available online at https://www.mdpi.com/2218-1989/9/5/82/s1, Table S1: Statistics of transcriptomics dataset, Table S2: Statistics of metabolomics and lipidomics dataset, Table S3: Upregulated gene sets in the LASCPC-01 cell line and SCNC patients, Table S4: Upregulated gene sets in the LNCAP cell line and prostate adenocarcinoma patients, Table S5: ChemRICH analysis of LASCPC-01 and LNCAP cell lines, Transcriptomics data were available at https://figshare.com/s/c2f67b643e0d207179b6.
